# Burden of stillbirths and associated factors in Yirgalem Hospital, Southern Ethiopia: a facility based cross-sectional study

**DOI:** 10.1186/s12884-020-03296-x

**Published:** 2020-10-06

**Authors:** Sintayehu Mengesha, Mesay Hailu Dangisso

**Affiliations:** College of Medicine and Health Sciences, School of Public Health, Hawassa University, Hawassa, Ethiopia

**Keywords:** Stillbirths, Pregnancy outcomes, Maternal health, Southern Ethiopia

## Abstract

**Background:**

Stillbirth is an adverse pregnancy outcome of public health importance causing considerable psychosocial burden on parents and their family. Studies on stillbirth are scarce in southern Ethiopia. An assessment of stillbirths and associated factors in health care settings helps in devising strategies for tailored interventions. Therefore, we assessed the burden of stillbirths and associated factors in Yirgalem Hospital, southern Ethiopia.

**Methods:**

A facility based cross-sectional study was conducted between 1 and 2015 and 30 July 2016. We randomly selected medical records of pregnant women from a hospital delivery registry. Bivariate analysis was employed to assess the association between independent and dependent variables using chi-square with significant p-value. Multivariate logistic regression was used to identify independent risk factors for stillbirths and to control for confounding variables.

**Results:**

Of 374 reviewed records of pregnant women, 370 were included for the study. The magnitude of stillbirths was 92 per 1000 births. Fifteen (44.1%) of fetal deaths occurred after admission to the hospital. In multivariate logistic regression, stillbirths were higher among low birth-weight babies (< 2500grams) (adjusted odds ratio (AOR): 10.70, 95% CI 3.18–35.97) than normal birth-weight babies (2500-<4000). Pregnant women who experienced a prolonged labour for more than 48 hours were 12 times (AOR: 12.15, 95% CI 1.76–84.12) more likely to have stillbirths than pregnant women without a prolonged labour. Pregnant women with obstetric complications were 18.9 times more likely to have stillbirths than pregnant women without obstetric complications. Similarly, pregnant women with at least two pregnancies were more likely to have stillbirths than pregnant women with less than two pregnancies (AOR: 4.39, 95% CI 1.21–15.85).

**Conclusions:**

We found a high burden of stillbirths in the study setting. Modifiable risk factors contributed to a higher risk of stillbirths; therefore, tailored interventions such as early identification and management of prolonged labour and obstetric complication at each level of health system could avert preventable stillbirths.

## Background

Stillbirth is an adverse pregnancy outcome of public health importance causing considerable psychosocial burden on parents and their family [1–3]. In 2015, the global stillbirth rate (SBR) was approximately 18·4 per 1000 births, 25.5% reduction from what was in 2000. Two-third of the world’s total stillbirths occurred in Southeast Asia and sub-Saharan Africa [4, 5]. Globally, the Every Newborn Action Plan advocates the target of achieving 12 or fewer stillbirths per 1000 births in every country by 2030 [5]. In resource poor settings, stillbirths are under studied and under reported despite their high burden and low reduction rates over years. This is because of various factors such as less attention given to the problem by health systems, inadequate reporting and poor access to quality obstetric care services [6]. Most stillbirths can be prevented by existing public health measures. However, the prevalence of stillbirths remains unacceptably high in resource poor settings despite the presence of high impact interventions. Stillbirths are classified as ante partum stillbirths and intra partum stillbirths depending on the time of the incident. Ante partum stillbirth occurs before the onset of labour, whereas intra partum stillbirth is the death of fetus after the onset of labour but before birth [7].

In resource-constrained settings, about half of stillbirths occur during labour or birth [1], and intra partum stillbirths are a commonly reported adverse birth outcome. Reports show that about 1.3 million intra partum stillbirths occurred in 2015 [8]. Intra partum stillbirths represent poor access to maternal care services and delays in getting appropriate obstetric care, particularly at health facilities. Intra partum stillbirth can be diagnosed with careful measurement of fetal heartbeat at onset of labour. In settings with poor access to skilled attendant during labour and poor practice of monitoring the fetal heartbeat, clinical indicators such as the skin appearance of the fetus (being macerated or unmacerated) help to identify intra partum stillbirths [5]. Thus, the unmacerated or fresh skin appearance can be used as an indicator of intra partum fetal death, while the macerated skin appearance could indicate stillbirths that occur before the onset of labour. Evidence shows that signs of skin maceration begin at 6–12 hours after fetal death although this measure could miss deaths occur at home and those who come after 12 hours due to delays in getting care and access to skilled attendance [9].

The burden of stillbirths varies between and within countries and the variations could be because of differences in the distribution of risk factors such as socio-demographic characteristics, fetal medical conditions, environmental exposures and psychosocial stressors [5, 10–12]. Maternal factors, for example, older age (above 35 years), obesity and smoking increase the risk of fetal death [3, 11]. Null parity, grand multiparity, obstructed labor, prolonged labour, placental abruption, placenta previa, preterm labor, premature rupture of membrane, and intrauterine growth restriction are common obstetric factors associated with an increased risk of stillbirths, particularly in resource poor settings [12–15].

Other maternal medical conditions such as thyroid diseases, cardiovascular disorders, asthma, kidney diseases, and diabetes also increase the risk of stillbirths [15–18]. Congenital anomalies of fetus [12], fetal-maternal hemorrhage [15, 19], and maternal infections such as malaria and syphilis also contribute to the risk of stillbirths in high burden areas [15].

In Ethiopia, the proportion of births attended by skilled attendants was only 28% [20], and the country was in the 5th place in number of stillbirths in the world in 2015 [5]. Moreover, the rate of stillbirth in Ethiopia was stable over decades without being declined with the average annual reduction rate (ARR) of three percent [21, 22]. The ARR of stillbirth in Ethiopia is lower than the reduction rate of some sub-Saharan African countries and the global ARR [5]. There are also variations in proportions of stillbirth and perinatal mortality within the country [20]. Studies from Ethiopia reported that the stillbirth rates ranged from 25/1000 births in Addis Ababa [21] to 85/1000 births in Amhara region [23]. A disproportionate burden of stillbirths in the country could be attributed to a poorly-equipped maternal health care services, poor access to and coverage of skilled attendance at birth, late referral, long distance to referral facilities, poor transport facility and poor quality of obstetric care services. Moreover, modifiable risk factors such as sever pre-eclampsia, maternal convulsion, placental abruption, cord accidents, lack of antenatal follow-up and low birth weight were also reported [3, 21].

Studies on stillbirth are scarce in southern Ethiopia and existing evidence focuses on neonatal and perinatal mortalities though stillbirths account for about half of perinatal deaths [6]. An assessment of the burden of stillbirths and associated factors in health care settings helps in devising strategies for tailored interventions in order to improve pregnancy outcomes [5]. In this study, we aimed to assess the magnitude of stillbirths and associated socio-demographic and obstetric factors in Yirgalem Hospital, southern Ethiopia.

## Methods

### Study setting

This study was conducted in Yirgalem General Hospital, which is located in Sidama Region, 310 km from Addis Ababa: the capital of Ethiopia. The hospital has a catchment population of 4.3 million. Mothers in labour are often admitted and managed in the maternity ward of the hospital. Majority of mothers in labour are referred from the neighboring district hospitals and health centers for various obstetric and medical reasons [28]. The total number of deliveries in one year period (from August 1, 2015 to July 30, 2016) in the hospital was reported to be 3000.

### Study design and period

A hospital based cross-sectional study was conducted by reviewing medical records of pregnant women who gave births in the hospital between August 1, 2015 and July 30, 2016.We selected the medical records of pregnant women in the study period randomly using medical record identification number (ID) from a delivery registry. The sample size used for the study was 374, which was computed using OpenEpi by considering the following assumptions: proportion of stillbirth in health care settings (50%), 5% margin of error, 95% confidence level and 10% for missing information or charts.

### Sampling technique and data collection procedure

First, we counted the number of deliveries in the hospital from a delivery registration book, which had IDs of pregnant women. A list of IDs was developed by registering medical record IDs of women admitted to a maternity ward in the hospital. Then, the IDs of the study subjects were selected randomly from the list using *random sample of cases* function in SPSS version 20.Then, we identified medical charts of pregnant women with the randomly selected IDs. Data on the socio-demographic, obstetric and medical history were collected from the maternal records (randomly selected medical charts) of the hospital. The independent variables collected from the maternal records were maternal age, place of residence, weight, height, marital status, educational level, occupation, hemoglobin level, blood pressure and laboratory tests for diabetes mellitus and malaria. Moreover, other obstetric variables such as antenatal follow-up, parity, gestational age, information about obstructed labor and preterm delivery, duration of labor, history of abortion, birth weight, and history of stillbirth and mode of delivery were collected. We used a structured checklist to collect the data and the checklist was pretested in 5% of maternity records of the previous years to check for consistency of information. The pretest data were not included in the study. The data were collected by trained nurses, and the data collected from the maternal records were checked for completeness and consistency and this was done by the principal investigator and the supervisor on a daily basis.

### Data management and analysis

The data were explored for errors, coded, entered, cleaned, and analysed by SPSS version 20. Descriptive statistics were used to summarize the data and bivariate analysis was employed to assess the association between independent and dependent variables. Multivariate logistic regression was used to identify independent risk factors for stillbirth and to control for confounding. Variables with p < 0.2 in bivariate analysis were included for multivariate logistic regression model. We used the cut-off point of p < 0.05 as a measure of statistical significance. Model diagnostics was done using Hosmer-Lemeshow Test and the goodness-of-fit statistic was considered adequate when a significance value was > 0.05.

## Results

Of 374 reviewed records of pregnant women, 370 (98.9%) were included for the study and 4 were excluded because of missing information. The mean age and the standard deviation (SD) of the study subjects were 23.9 and 4.6 respectively. Majority, 314 (89.1%) pregnant women were in the age group of 20–34 years. There was no participant under age of 16 years. A higher proportion, 273 (74%) pregnant women were from rural settings; 191(51.6%) were directly admitted to the hospital; 179 (48.4%) were referred from other facilities (Table 1).

### Medical conditions of the study subjects

Majority, 369 (99.7%) of pregnant women had no history of chronic illness except one woman with a history of epilepsy. Similarly, 365 (98.6%) of pregnant women had no history of infection at the time of admission. Only twenty-two (5.9%) women had protein in urine. However, 312 (84.3%) of them had no laboratory result of the urine test. Twenty- seven (7.3%) of women had anemia (Hgb < 11 g/dl), and the mean Hgb level was 12.74 g/dl (SD ± 2) (Table 1).

**Table 1:** Socio-demographic factors and medical conditions of study subjects (n = 370) at Yirgalem General Hospital in Sidama Region, Ethiopia, between August 1, 2015 and July 30, 2016

Category	Frequency (%)
**Maternal age (in year)**	
< 20	36 (9.7)
20–34	314 (84.9)
≥ 35	20 (5.4)
**Residence**	
Rural	273 (73.8)
Urban	97 (26.2)
**Admission status**	
Referred	179 (48.4)
Direct admission	191 (51.6)
**History of chronic illness**	
Yes	369 (99.7)
No	1 (0.3)
**Infection**	
Malaria	1 (0.3)
HIV	1 (0.3)
Chorioamnitis	3 (0.8)
None	365 (98.6)
**Proteinuria**	
Yes	22 (5.9)
No	36 (9.7)
Not done	312 (84.3)
**VDRL**	
Non-reactive	2 (0.5)
Not done	368 (99.5)
**Diabetes**	
Yes	1 (0.2)
No	369 (99.8)
**Hemoglobin level**	
< 10.99 g/dl	27 (7.3)
≥ 11 g/dl	335 (90.5)
Unknown	8 (2.2)

### Obstetric characteristics of women

Ninety-eight percent (363 pregnant women) had antenatal care follow up, and 20 (5.4%) of them had a history of abortion. More than half, 201(54.3%) of the pregnant women were primgravida and 209 (56.5%) were nullpara (para 0). About half, (51.9%) of women did not know the gestational age of their pregnancy at time of admission, while 138 (37.3%) of them were within the gestational age ranging from 37 to 42 weeks. The median duration of labor was 14 hours (interquartile range of 10–20). A higher proportion of women, 193 (52.2%) had a prolonged labor lasting more than 12 hours. Majority, 286 (77.3%) women delivered without being assisted by instruments such as vacuum extractor or obstetric forceps, whereas 58 (15.7%) delivered by cesarean section. Of 370 deliveries, 312 (84.3%) of the babies had normal weight (weighing 2500 to < 4000 grams), while 29 (7.8%) of them were 4000 grams and above. Twenty (5.4%) of the women had hypertensive disorders of pregnancy; 29 (7.8%) had premature rupture of membrane (PROM); 16 (4.3%) had obstructed labor; 10 (2.7%) had a cord accident, and 8 (2.2%) experienced ante-partum hemorrhage (Table 2).

**Table 2:** Obstetric factors and birth outcomes of women (n = 370) at Yirgalem General Hospital Sidama Region, Ethiopia, August 1, 2015-July 30, 2016

Characteristics	Frequency (%)
**ANC**	
Yes	363 (98.1)
No	1 (0.30)
Unknown	6 (1.60)
**History of abortion**	
Yes	20 (5.40)
No	349 (94.3)
Unknown	1 (0.30)
**History of previous stillbirth**	
Yes	2 (0.50)
No	367 (99.20)
Unknown	1 (0.30)
**Gravidity**	
I	201 (53.30)
II-IV	124 (33.50)
≥V	45 (12.20)
**Parity**	
0	209 (56.50)
I	64 (17.30)
II-IV	70 (18.90)
≥V	27 (7.30)
**GA (n weeks)**	
< 37	30 (8.10)
37–42	138 (37.30)
≥ 42	10 (2.70)
Unknown	192 (51.90)
**Birth weight**	
< 2500	27 (7.30)
2500–3950	312 (84.3)
≥ 4000	31 (8.40)
**Duration of labour**	
≤ 12 hours	169 (45.7)
13–48 hours	182 (49.2)
> 48 hours	11 (3.0)
Unrecorded	8 (2.2)
**Mode of delivery**	
Spontaneous vaginal delivery	286 (77.30)
Caesarian section	58 (15.70)
Instrumental delivery	20 (5.40)
Laparotomy	4 (1.10)
Destructive delivery	2 (0.50)
**Hypertension**	
Yes	20 (5.4)
No	350 (94.6)
**Antepartum hemorrhage**	
Yes	8 (2.2)
No	362 (97.8)
**Premature rupture of membrane**	
Yes	28 (7.6)
No	342 (92.4)
**Obstructed labour**	
Yes	16 (4.3)
No	354 (95.7)
**Uterine rapture**	
Yes	4 (1.1)
No	366 (99.9)
**Cord accident**	
Yes	10 (2.7)
No	360 (97.3)
**Birth outcomes**	
Dead	34 (9.20)
Alive	336 (90.80)
**Skin appearance**	
Fresh	22 (64.70)
Macerated	10 (29.40)
Not recorded	1 (2.90)
**Fetal heart beat at admission among stillbirths**	
Positive	15 (44.10)
Negative	19 (55.9)

### Magnitude of still birth and associated factors

In this study, the magnitude of stillbirth was 92 per 1000 births. Fifteen (44.1%) fetal deaths occurred after admission to the hospital. Pregnant women with a prolonged labour had 18 (53%) of fetal deaths. In multivariate analysis, the odds of stillbirths was 10.7 times higher among low birth-weight babies (< 2500grams) (adjusted odds ratio (AOR): 10.70, 95% CI 3.18–35.97) than normal weight babies (2500-<4000). The odds of stillbirths was 12.15 times higher among women with a prolonged labour for more than 48 hours (AOR: 12.15, 95% CI 1.76–84.12) than women without a prolonged labour. The odds of stillbirths was 18.9 times higher among women with obstetric complications than women without obstetric complications. Similarly, the odds of stillbirths was four times higher among women with at least two pregnancies (AOR: 4.12, 95% CI 1.40–12.3) than women with less than two pregnancies (Table 3). Maternal anemia and mode of admission were associated with stillbirths in bivariate analysis; however, the association was not significant in multivariate analysis (Table 3). Parity was excluded from the final model because of its multicollinearity effect. We did a model diagnostic test using Hosmer-Lemeshow Test and the goodness-of-fit statistic had a significance value of > 0.05, and the model has adequately described the data.

**Table 3:** Multivariate analysis results for factors associated with stillbirth in Yirgalem General Hospital, August 1, 2015-July 30, 2016

Variables	Dead (%)	COR (95% CI)	AOR (95% CI)	*P*-value
**Gravidity**				
1	12 (6.0)	1.00	1.00	
2–4	14 (11.3)	2.0 (0.90–4.49)	4.12 (1.40–12.3)	0.011
> 4	8 (17.8)	3.41 (1.30–8.91)	4.39 (1.21–15.85)	0.024
**Parity**				
0	12 (5.7)	1.00	1.00	
1	39 (4.7)	0.81 (0.22–2.96)		
2–4	15 (21.4)	4.48 (1.98–10.12		
> 4	4 (14.8)	2.86 (0.85–9.59)		
**Mode of admission**				
Self-admitted	10 (5.2)	1.00	1.00	
Referred from other facilities	24 (13.4)	2.80 (1.3–6.04)	1.94 ( 0.75–5.01)	0.171
**Maternal anemia**				
No	28 (8.4)	1:00	1:00	
Yes	6 (22.2)	3.13 (1.17–8.40)	1.87 ( 0.45–7.81)	0.390
**Presence of obstetric Complications**				
No	5 (1.9)	1:00	1:00	
Yes	29 (28.2)	20.54 (7.68–54.9)	18.94 (6.43–55.81)*	< 0.001
**Duration of labour**				
≤ 12 hours	16 (9.5)	1.00	1.00	
13–48 hours	13 (7.1)	0.74 (0.34–1.58)	1.32 (0.49–3.56)	0.581
> 48 hours	5 (45.5)	7.97 (2.19–29.1)	12.15 (1.76–84.12)*	0.011
**Weight of baby**				
2500-<4000 grams	21 (6.7)	1.00	1.00	
< 2500 grams	10 (37.0)	8.15 (3.32-20.0)	10.70 (3.18–35.97)*	< 0.001
≥ 4000	3 (9.7)	1.49 (0.42–5.29)	0.77 (0.15–3.88)	0.748

*Statistically significant at *P* < 0.05

## Discussion

In this facility based study, we found a high burden of stillbirths with 92 deaths per 1,000 births. Low birth weight, obstetric complications, prolonged labour and gravidity of at least two were associated with a higher risk of stillbirths. The finding of this study was higher than previous studies conducted in Hawassa [24], Gondar [23] and Tikur Anbessa [21] hospitals in Ethiopia. The variations could partly be explained by differences in study setting and quality of obstetric care services in the facilities. Thus, aforementioned studies were conducted in referral hospitals which have better obstetric care facilities and skilled health workforce, whereas our study setting was a general hospital with fewer obstetricians and majority of cases were referred from peripheral primary health care units with poor facilities, which provide obstetric services for a predominantly rural population. The proportion of stillbirths in our finding was also higher than the global estimates of stillbirth for Ethiopia [25, 26] and other studies from developing countries such as Bangladeshi [27] and India [28]. This variation could be partly explained by differences in study settings and methods because the present study was a hospital based study unlike other community based studies from developing countries. Moreover, socio-demographic, economic, health systems and cultural factors could contribute to the observed differences in the burden of stillbirths. However, our finding was almost consistent with a study from Pakistan [13].

In our study, fetal death after admission (44.1%) was higher than a study from Hawassa (15%) [24] and Tikur Anbesa (20%) [21] hospitals in Ethiopia. This variation could be due to a varying level of delays in getting care which results in significant fetal jeopardy and complications or could be due to sub-optimal management of labour and delivery. Similarly, majority (64.7%) deaths were fresh (unmacerated), which indicate that most deaths were peripartum and the finding is comparable to the multi-country study finding from Kenya, Zambia, India, Pakistan, Guatemala and Argentina [29]. Intra partum deaths are unacceptable because they are considered preventable, and could be associated with delays in getting quality care around the time of birth and poor management of labour at health facilities [29]. However, the intra partum stillbirth estimate based on skin appearance (macerated or not being macerated) could underestimate the intra partum stillbirth rates. This could be because fetal deaths could occur at home before pregnant mothers visit a health facility. Improving access to quality intrapartum care is crucial for reducing intrapartum stillbirths and could also contribute to improvements in pregnancy outcomes. This information helps in devising interventions for reducing stillbirths.

In this study, low birth weight had 10.7 times higher risk of stillbirths compared to the risk of stillbirths among normal birth weight babies. This could be due to the fact that low birth weight fetuses are more likely to be immature and could not tolerate intrauterine asphyxia because of different physiological reasons. This finding is consistent with the findings of studies conducted in Gondar, Ethiopia [3], Pakistan [30] and a systemic review report of low and middle income countries [15]. Interventions focusing on preventing intrauterine growth restriction and low birth weight should be devised to reduce the problem. In our study, obstetric complications (hypertensive disorders of pregnancy, antepartum hemorrhage, premature rupture of membrane, obstructed labour, uterine rapture and cord accident combined) were significantly associated with stillbirths, and this finding was consistent with studies from elsewhere [3, 12, 21]. Early identification and management of mothers with obstetric complication could reduce burden of preventable stillbirths.

Mothers with a prolonged labour for more than 48 hours were 12 times more likely to develop stillbirths than those without a prolonged labour. Poor progress of labour is often attributed to obstructed labour which is a common risk factor for stillbirths. Evidence shows that prolonged labour can be attributed to poor uterine action, malpresentation or large fetal head and obstructions in pelvis or birth canal [3]. A prolonged labour could affect maternal health condition and contributes to asphyxia and fetal distress, which affects the fetal survival. Improving access to quality maternal health care services and early detection and management of labour are of paramount importance to reduce stillbirths attributed to a prolonged labour.

One of the limitations of this study was its cross-sectional nature, and there would be some unmeasured socio-economic and demographic variables which could have been associated with stillbirths. Variables such as time of ANC initiation and ANC provider were not included because the variables were not recorded in delivery registration book. However, the study provides valuable information about the burden of stillbirths and modifiable risk factors that need tailored interventions at health facility settings.

## Conclusions

The study has shown a high proportion of stillbirths in the study settings which require immediate action. A high proportion of intrapartum stillbirths question the quality of obstetric care since intrapartum stillbirths are considered preventable. Therefore, devising focused high impact interventions, such as early identification and management of pregnant women with prolonged labour and obstetric complications, and addressing modifiable risk factors at each level of health system could avert preventable stillbirths.

## Data Availability

The datasets used and/or analysed during the current study are available from the corresponding author on reasonable request.

## Abbreviations

ANC: Antenatal care
AOR: Adjusted odds ratio
ARR: Annual reduction rate
CI: Confidence interval
COR: Crude odds ratio
Hgb: Haemoglobin
HIV: Human immunodeficiency virus
ID: Identification number
PROM: Premature rupture of membrane
SBR: Stillbirth rate
SD: Standard deviation
VDRL: Veneral Diseases Research Laboratory
